# A Universal Digital Lock-in Amplifier Design for Calibrating the Photo-Detector Responses with Standard Black-Bodies

**DOI:** 10.3390/s23218902

**Published:** 2023-11-01

**Authors:** Zheyi Yao, Jingpeng Pan, Chang Yu, Zhewen Yuan, Qian Chen, Xiubao Sui

**Affiliations:** 1 School of Electronic and Optical Engineering, Nanjing University of Science & Technology, 200 Xiaolingwei, Nanjing 210094, China; zheyi@njust.edu.cn (Z.Y.);; 2 The Jiangsu Key Laboratory of Spectral Imaging & Intelligent Sense, Nanjing 210094, China

**Keywords:** lock-in amplification algorithm, IIR filter, DDS, CORDIC algorithm

## Abstract

The lock-in amplifier (LIA) is widely utilized to detect ultra-weak optical periodic signals based on the phase-sensitive and enhanced detecting theory. In this paper, we present an all-digital and universal embedded LIA platform that accurately and conveniently describes the spectrum generated by standard black bodies at various temperatures with different optical detectors. The proposed design significantly reduces the complexity and cost of traditional analog LIAs while maintaining accuracy. The LIA components are implemented using a single field programmable gate array (FPGA), offering flexibility to modify parameters for different situations. The normalized mean-square error (NMSE) of the captured spectra in the experiments is within 0.9% compared the theoretical values.

## 1. Introduction

There are various methods have been proposed to capture the precise optical spectra from heavy noise and conduct accurate measurements, including lock-in amplifiers (LIAs), signal averaging, boxcar integrators, and correlators [1,2,3]. These methods share a similar philosophy, using differential strategies to reduce the noise bandwidth and amplify the desired signals simultaneously. Among the above methods, LIAs are particularly suitable for suppressing both the environmental and intrinsic detector noise to extract the desired signal. LIAs have been widely applied in diverse applications [4,5,6], including gravitational wave detection, quantum phenomenon demodulation, and imaging.

Since the LIA utilizes the coherence theory [7] to enhance the ultra-weak signal, its core is the phase coherence theory. The LIA utilizes the signal’s time dependence, sometimes termed as the down-mixing or heterodyne/homodyne detection [8], to enhance the weak signals by performing phase-sensitive detection, where the reference signal has the same modulating frequency as the desired one. Through the LIA, the desired signal components, both the amplitude and initial phase, can be extracted. Additionally, with the increasing development of integrated circuits, all-digital LIAs have become advantageous over analog designs in terms of the size, cost, and flexibility. Over the past few decades, field-programmable gate arrays (FPGAs) have been proven as a great prototyping platform for all-digital integrated circuit demonstrations [9,10]. Equipped with a parallel processor, multiplier unit, and direct digital synthesizer (DDS) [11] in the embedded platform, FPGAs enable designers to achieve all-digital LIA designs with higher speed, greater flexibility, and smaller size than traditional analog LIAs [12,13]. In addition, the proposed design in this paper contains extra functions, such as auto-phase alignment, oversampling, and dual-phase, which are applicable for accurate and flexible applications in the future.

To demonstrate the LIA ability to capture precise optical spectra of various standard black-bodies, some experimental measurements were conducted based on a single printed circuit board (PCB). By combining the circular variable filter (CVF) with optoelectrical detectors, such as MCT (HgCdTe) and InSb, the various blackbody spectra can be captured. Compared with the theorical values, the normalized mean-square error (NMSE) of simulations and measuring spectrum experiments are less than 0.826% and 0.9%, respectively, which confirms the system accuracy of the proposed design.

## 2. The Theory and Principle

### 2.1. Overview

**Figure 1:**
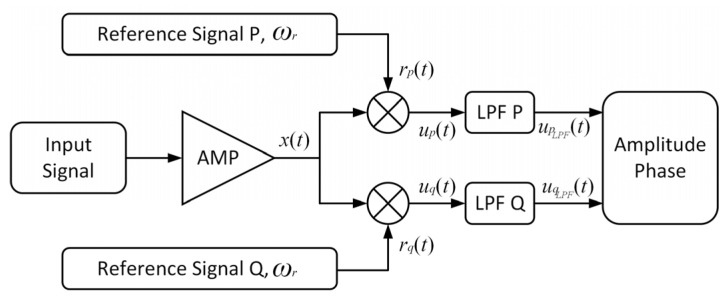
Structure diagram of phase-locked amplifier: AMP, amplifier, LPF, low-pass filter.

Figure 1 shows the basic structure of the two-channel lock-in amplifier (LIA), which consists of the signal channel, in-phase and quadrature reference channels that are termed as P and Q, both of which contain the phase sensitive detection (PSD) part, low pass filter (LPF). The PSD function is achieved with a mixer in the block diagram by multiplexing the orthogonal reference signals, which has π/2 constant phase difference between each other with the input signal, and the LPFs utilized in two channels share the same parameters, such as the suppressing ratio, passband, and stopband. Then, the amplitude and phase of the original signal can be extracted, and the mathematical relation is presented in detail as following.

Suppose there are the following signals:

(1) $$x\left(t\right)=Asin\left(\omega_{s}t+\varphi_{s}\right)+N\;r_{p}\left(t\right)=Bsin\left(\omega_{r}t+\varphi_{r}\right)\;r_{q}\left(t\right)=Bcos(\omega_{r}t+\varphi_{r})$$

where the x(t) is the original electronic signal captured by the optoelectronic detector with the noise N that is with unknown form and frequency distribution; the $r_{p}\left(t\right)$ and $r_{q}\left(t\right)$ are the reference signals for two channels; A, B, $\omega_{s}$, $\omega_{r}$, $\varphi_{s}$, $\varphi_{r}$, and t are the amplitudes, angular speeds, the initial phases of input and reference signals of the input signal and reference signals, and time, respectively. It is clear that there is a constant phase shift between two reference channels.

Then, the signal after the multiplication of the in-phase part can be expressed as:

(2) $$\begin{array}{ll} u_{p}\left(t\right) & =x\left(t\right)\times r_{p}\left(t\right)\\ & =\frac{AB}{2}\{\cos\left[\left(\omega_{s}-\omega_{r}\right)t+\left(\varphi_{s}-\varphi_{r}\right)\right]\\ & \;\;\;\;\;\;\;\;\;\;\;\;\;\;\;-\cos\left[\left(\omega_{s}+\omega_{r}\right)t+\left(\varphi_{s}+\varphi_{r}\right)\right]\}+Nr_{p}\left(t\right) \end{array}$$

And the quadrature part signal is similar with the in-phase one. It can be seen that the output signal consists of a high frequency component with an angular speed of $\omega_{s}+\omega_{r}$ and a low frequency component with the angular speed of $\omega_{s}-\omega_{r}$. The noise part, $Nr_{p}(t)$, is modulated by the referencing frequency, $\omega_{r}$. If $\omega_{s}=\omega_{r}$, the low frequency part will directly become DC signal, and then through the ideal low-pass filter (LPF), the output signal, $u_{p_{LPF}}(t)$, is:

(3) $$u_{p_{LPF}}(t)=\frac{AB}{2}cos\left(\varphi_{s}-\varphi_{r}\right)+{\left[Nr_{p}\left(t\right)\right]}_{LPF}$$

in which ${\left[Nr_{p}\left(t\right)\right]}_{LPF}$ is the modulated noise in the LPF passband from the in-phase channel, and the output signal for the quadrature part is termed as $u_{q_{LPF}}(t)=\frac{AB}{2}sin\left(\varphi_{s}-\varphi_{r}\right)+{\left[Nr_{q}\left(t\right)\right]}_{LPF}$.

It can be seen from the above formula that if the phase difference between the two signals is constant, the output signal is proportional to the amplitude of the input signal; thus, the noise can be limited within the LPF narrow passband. To suppress the noise effectively in the narrow bandwidth, an appropriate filter ought to be selected. Thus, both the amplitude and phase can be captured from the in-phase and quadrature outputs $u_{p_{LPF}}(t)$, $u_{q_{LPF}}(t)$, which are sensitive to the amplitude and phase of x(t).

### 2.2. The Design of the Digital Lock-in Amplifier

Currently, the digital LIAs are mostly built using Microcontroller Units (MCUs), Digital Signal Processors (DSPs) [14], FPGAs [15,16], and Personal Computers (PCs). Compared to analog devices, the quantified signals of digital circuit platforms are more robust and flexible, allowing them to overcome issues caused by temperature drift, random noise sources, and relatively poor stability. Additionally, due to the repeatability of their design, digital LIAs significantly reduce the cost of circuit replacement and have become the mainstream technology for LIA.

**Figure 2:**
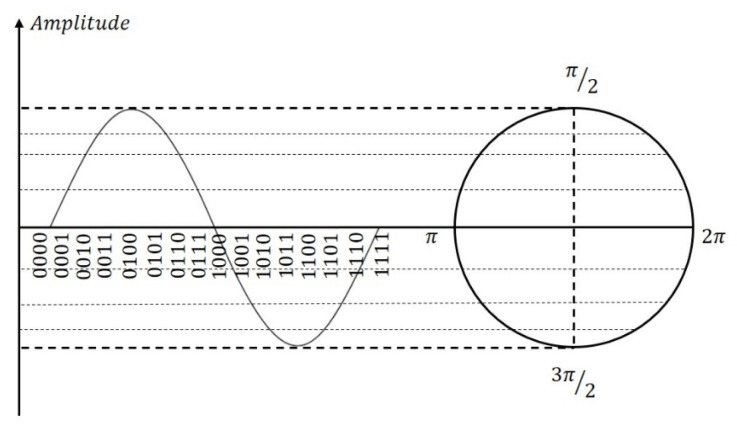
Corresponding relationship between phase word and amplitude of trigonometric function.

To generate artificially controlled referencing waves, the digital platform utilizes the DDS, in which the corresponding relationship between the phase and amplitude of the referencing signal that is a 4-bit digitalized number is depicted in Figure 2. The time it takes for the circle to complete one full rotation determines the frequency of the sine wave, namely:

(4) f=fclk2Bnco

Among the above formula, $B_{nco}$ is the phase accumulation word width, indicating the number of bits of the phase point, and $f_{clk}$ is the main frequency of the digital system.

**Figure 3:**
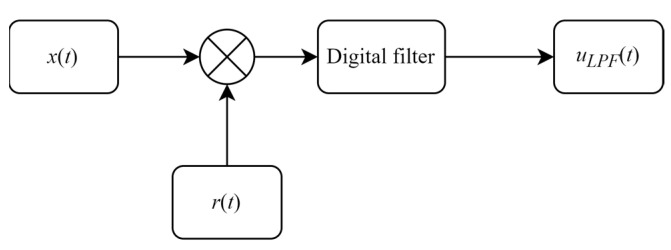
The phase sensitive detector with digital filter.

The phase-sensitive detector plays a crucial role in phase identification and can be treated as a phase comparator that compares the differences between the reference and the original signals, then generating the phase error between them [17]. When the input signal and the reference signal have the same frequencies but a constant phase difference, as shown in Equation (3), the phase and original amplitude can be extracted from the output signal $u_{LPF}(t)$, which consists of $u_{p_{LPF}}(t)$ and $u_{q_{LPF}}(t)$. Generally, in the digital system, the PSD can be regarded as being composed of a multiplier, followed by the low-pass filter (LPF), as shown in Figure 3.

**Figure 4:**
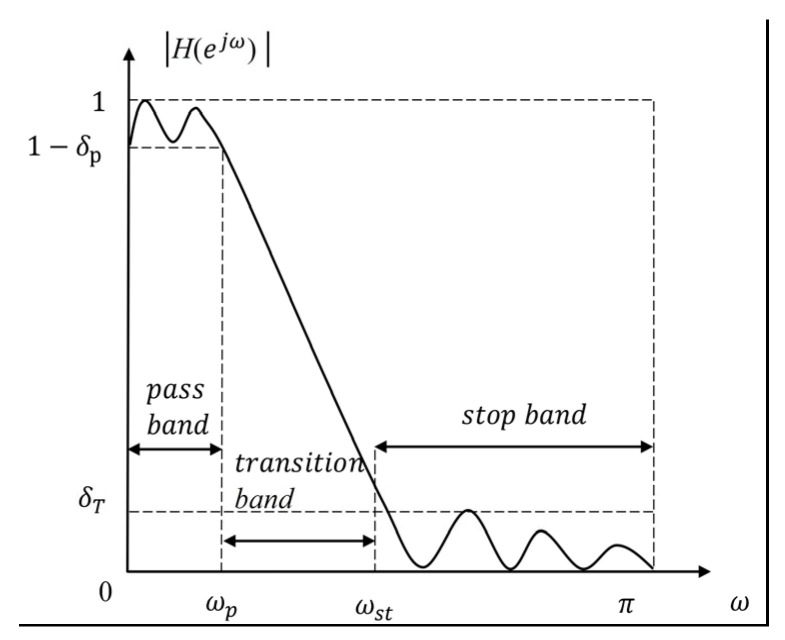
The amplitude-frequency response diagram of digital low-pass filter, where $\omega_{p}$ represents the passband cutoff frequency, $\omega_{st}$ represents stopband cut-off frequency, $∆\omega =\omega_{st}-\omega_{p}$ is the transition band, $\delta_{p}$ represents the passband ripple, and $\delta_{T}$ is the stop band ripple, respectively.

For the lock-in amplifier, the LPF is utilized to suppress both the system noise and the high frequency modulated signals mentioned above. Considering that the modulated signal is similar to the DC signal in Equation (3), the low-pass filter with a narrow passband is selected to extract the desired signal. Furthermore, the narrower the passband, the less noise left for the amplitude and phase calculations. The general amplitude-frequency response of the LPF is illustrated in Figure 4.

Generally, there are two categories of digital filters [18]: the finite impulse response (FIR) and infinite impulse response (IIR). Even though the FIR filters are inherently stable, IIR filters can achieve better filtering effects with lower orders, which means less resources and time delays in the embedded digital systems. In addition, only linear magnitude linearity is desired in the optical spectrum capturing, whereas the nonlinearity phase would not affect the spectrum measuring. However, to ensure stability when implementing an IIR digital filter in the LIA, the selecting poles should be designed within the unit circle.

Compared with other types IIR filters, the Chebyshev type II filter does not contain any amplitude fluctuation in its passband, making it suitable for the spectrum capturing applications.

The system function *H*(*z*) of the direct IIR filter can be expressed as:

(5) Hz=YzXz=∑i=0Lbiz−i1−∑i=1Laiz−i

in which, $Y\left(z\right)$ and $X\left(z\right)$ are the *Z* transforms of y(n) and x(n), respectively.

**Figure 5:**
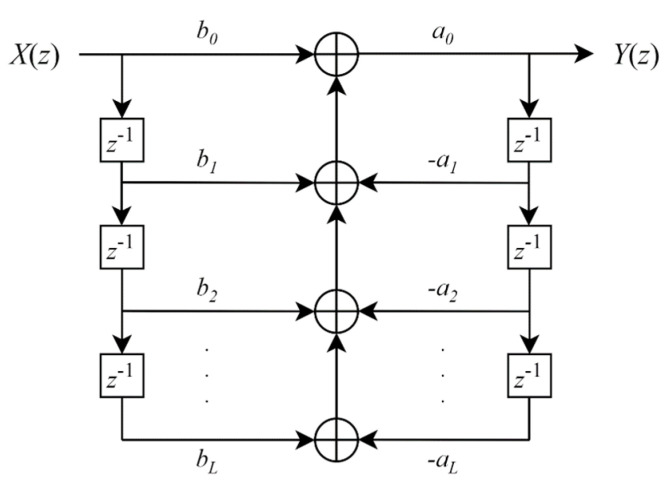
The schematic diagram of the low-pass IIR filter.

For the *L*-th order IIR filter, its schematic diagram can be obtained by the graphical description of Equation (5), as shown in Figure 5 below.

For the digital signal processing, it is often necessary to solve trigonometric function values and modulus values. The coordinated rotation digital computer (CORDIC) algorithm [19,20,21] is a hardware-efficient iterative method that uses the rotations to calculate a wide range of elementary functions to achieve the above tasks. In essence, the CORDIC algorithm takes a successive approximation of mathematical calculation. Since the basic operation unit of the algorithm only includes shifters and adders, the algorithm is simple and efficient in the digital system.

**Figure 6:**
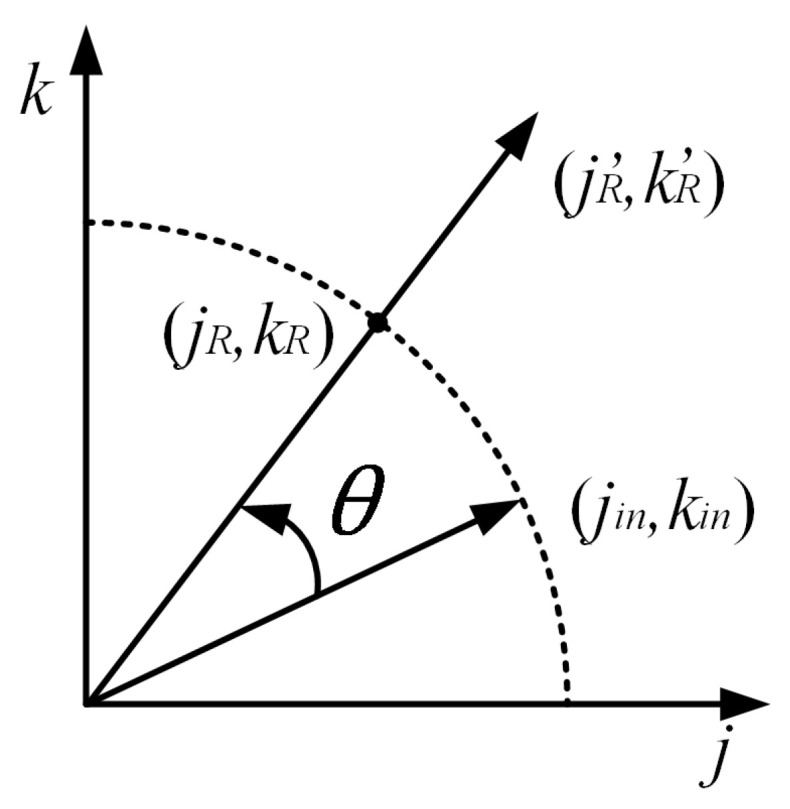
The CORDIC algorithm vector rotation diagram with rotating the input vector by θ.

The basic principle of the CORDIC algorithm is shown in Figure 6.

The coordinate transformation relation between two vectors is:

(6) $$\left\{\begin{array}{c} j_{R}=j_{in}cos\theta -k_{in}sin\theta \\ k_{R}=k_{in}cos\theta +j_{in}sin\theta \end{array}\right.$$

The pseudo-rotation equation can be obtained by dividing the two sides by cosθ, namely:

(7) $$\left\{\begin{array}{c} {j_{R}}^{\prime }=\frac{j_{R}}{cos\theta }=j_{in}-k_{in}tan\theta \\ {k_{R}}^{\prime }=\frac{k_{R}}{cos\theta }=k_{in}+j_{in}tan\theta \end{array}\right.$$

At this time, the rotation angle is correct, but the modulus of the vector changes.

The essence of the CORDIC algorithm is to rotate the coordinate $\left(j_{in},k_{in}\right)$. Each rotation is fixed angle $\theta_{i}$, and the rotation direction is $d_{i}=sign(k_{i})$. The goal of rotation is that the ordinate $k_{i}$ is close to 0. When *N*-step iteration is carried out, there is an anti-tangent angle.

(8) $$\theta =\sum_{i=0}^{N-1}d_{i}\theta_{i}$$

**Figure 7:**
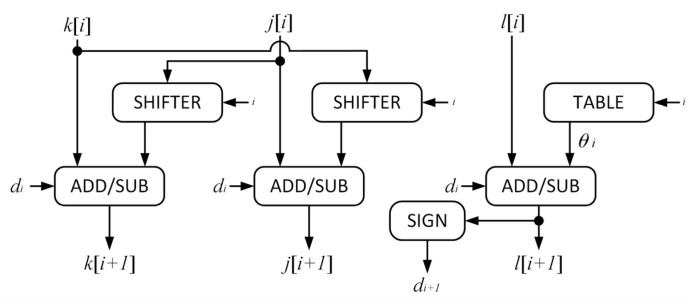
The *i*-th single iteration of CORDIC algorithm implementation. SHIFTER, phase shifter; ADD/SUB, adder or subtractor that contains conditional complementor; SIGN, sign bit capture.

In order to simplify the calculation process [22], the CORDIC algorithm uses a series of small rotation angles, denoted as $\theta_{i}$, to satisfy the equation ${tan\theta }_{i}=2^{-i}$, which allows for multiplication using simple shifting operations. This simplification transforms the original algorithm into an iterative shift-addition algorithm. The iterative equation is as follows:

(9) $$\left\{\begin{array}{c} j[i+1]=j[i]+2^{-i}d_{i}k[i]\\ k[i+1]=k[i]-2^{-i}d_{i}j[i]\\ l[i+1]=l[i]+d_{i}\theta_{i} \end{array}\right.$$

in which l[i] and $d_{i}$ are a template parameters, and

(10) $$d_{i}=\left\{\begin{array}{c} +1,l[i]\geq 0\\ -1,\;l[i]<0 \end{array}\right..$$

and Figure 7 shows the implementation of a single iteration in the CORDIC algorithm:

Suppose that the modulus of the vector needs to be maintained, it can be achieved by adding the rotation compensation factor K, which keeps constant after *N* iterations, as:

(11) $$K=\prod_{i=0}^{N-1}cos\theta_{i}=\prod_{i=0}^{N-1}\sqrt{\frac{1}{1+2^{-2i}}}$$

For the imbedded system, to simplify the calculating progress, the K serves as the initial value of j, $j\left[0\right]=K$, and is saved at the digital memory.

### 2.3. Spectrum Response Capturing Setup

**Figure 8:**
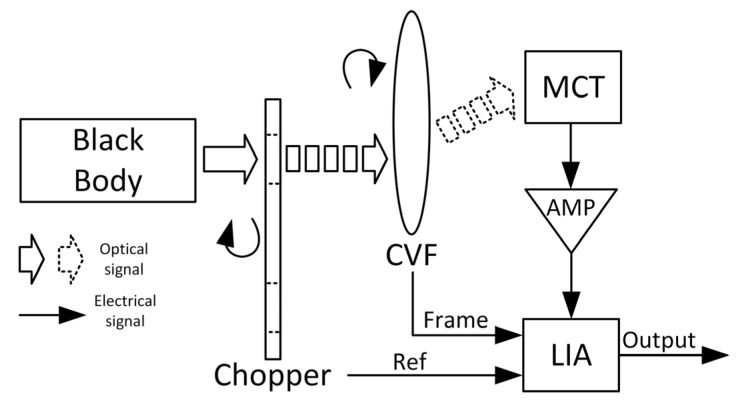
The setup of black body spectrum capturing. CVF, circular variant filter; MCT, HgCdTe detector; AMP, amplifier; LIA, locked-in amplifier.

To accurately capture the optical spectrum emitted by a black body, the experimental setup shown in Figure 8 is utilized. The setup includes a chopper, a circular variable filter (CVF), and an MCT detector to modulate the radiance from the source, select the corresponding wavelengths from the electromagnetic spectrum, and convert the radiant signal to an electrical one. However, the gains of the MCT and InSb photodetector are not sufficient to convert the radiance to the processing range of the LIA, so an additional amplifier (AMP) is introduced. The LIA requires referencing sync signals from both the chopper and CVF to extract the spectrum precisely. Finally, by combining the LIA output with the CVF wavelength index, accurate black body spectra can be obtained at various temperatures.

## 3. The Simulations and Experiments

### 3.1. Simulations for LIA Design

To judge the performance and stability of the proposed digital LIA, the MATLAB is implemented to simulate the proposed design with the parameters, as shown in Table 1, in which the chopper rate is set as 1000 Hz.

The noise signal is generated by using the rand function, then multiplied by signal to noise ratio (*SNR*), which utilizes the dB unit and is defined as:

(12) $$SNR=20\log_{10}\frac{A}{N}$$

in which, A is the original amplitude, and N is the root mean square of the noise.

For the LPF part, an IIR filter with Chebyshev type II, whose coefficients are set in advance, is utilized to process the mixed signals.

**Figure 9:**
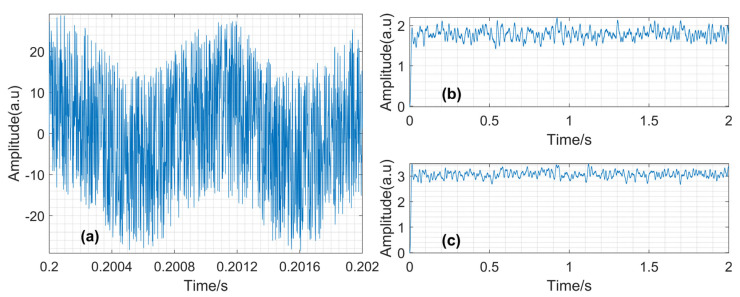
The simulation results. (**a**) Mixed input signal, x(t), with SNR = −10 in two periods; (**b**) Output signal from in-phase channel, $u_{p_{LPF}}(t)$; (**c**) Output signal from quadrature channel, $u_{q_{LPF}}(t)$.

The mixed input signal of the original signal and noise, or x(t), and the raw lock-in output signals from the in-phase and quadrature channels after phase locking, or $u_{p_{LPF}}(t)$ and $u_{q_{LPF}}(t)$, are shown in Figure 9.

**Figure 10:**
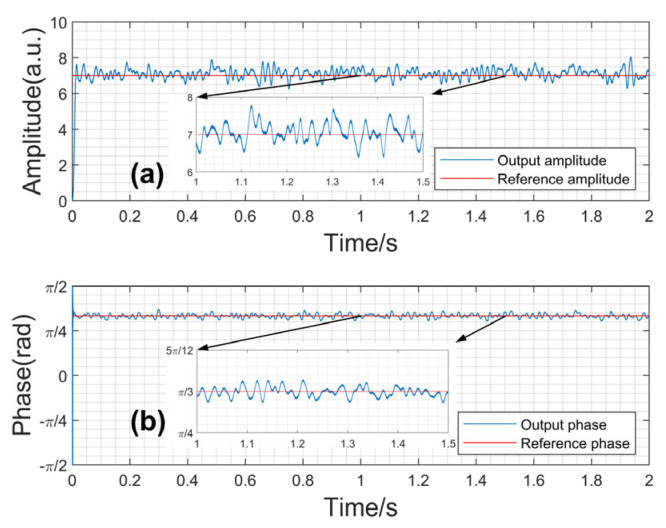
(**a**) The output amplitude and the reference amplitude. (**b**) The output phase and the reference phase.

The comparison between the output phase/amplitude and the reference phase/amplitude is shown in Figure 10.

The performance of the designed digital LIA in accurately detecting the amplitude and phase information of the input signal is demonstrated in Figure 10. The simulating results show that the measurement accuracies of amplitude and phase have improved compared to the conventional lock-in amplification algorithm. To quantify the average error, the Normalized Mean-Square Error (*NMSE*) is used, which takes the range of the data into account, and evaluates the accuracy of the prediction model. A smaller NMSE value indicates better accuracy in describing experimental data. The formula for NMSE is:

(13) $$NMSE=\frac{\sum_{i=1}^{n}{\left(\bar{y_{i}}-y_{i}\right)}^{2}}{\sum_{i=1}^{n}y_{i}^{2}}\times 100\%$$

where *n* is the number of samples, $\bar{y_{i}}$ is the average of sample *y*.

The NMSE is calculated to evaluate the accuracy of measurement results as shown in Table 2.

The value range of NMSE is [0,+∞]. The closer the value is to 0, the smaller the error of measurement results. The NMSE of phase and amplitude measurement results are both less than 0.01, therefore, the measurement results are relatively accurate.

### 3.2. Experiments with LIA

To illustrate the actual effect of the lock-in amplification algorithm, a hardware platform has been completed.

**Figure 11:**
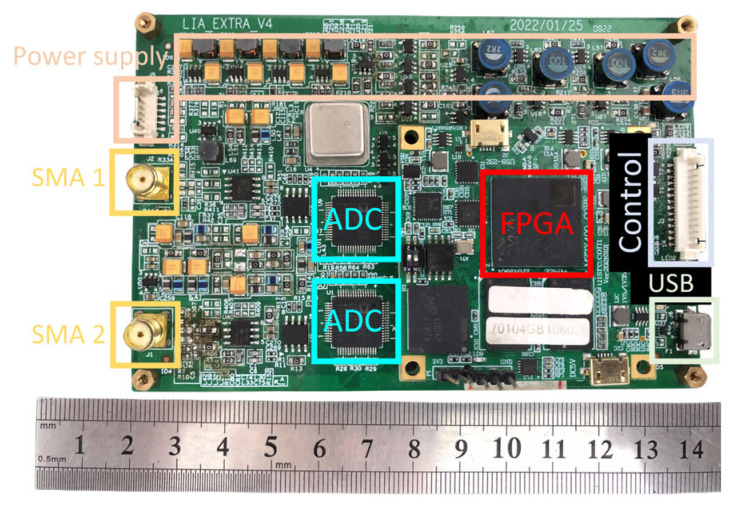
The LIA PCB platform.

The development board used in the experiment is equipped with the Zynq-7000 series chip, XC7Z-0710 of Xilinx Company. The circuit board has two-channel SMA inputs, using a high performance, 24-bit ADC, AD7760. The pre-amplifier circuit obtains the input photoelectric signal through the SMA interfaces. The proposed LIA design is shown in detail in Figure 11.

**Figure 12:**
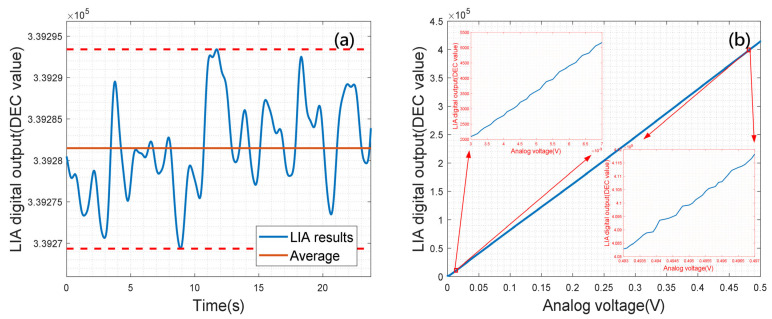
The linear response experimental results. (**a**) Single voltage measuring results with 0.4000 Vpp for 25 s; (**b**) Amplitudes measuring results with input amplitude ranging from 0 to 0.5 Vpp.

In the experimental setup, the desired parameters and embedded resources of the FPGA project are shown in Table 3. The original signal is provided to the LIA PCB board through the SMA connector, then the ADC that has a 625 k samples/s sampling rate is utilized to convert such a signal into a digital one to the FPGA, in which for the proposed digital LIA project, the processed values are finally sent out to the computer by the USB connector, with a 40,000 results/s processing rate. For the inner data of the FPGA project, the AXI Bus is utilized to connect the modules. An arbitrary waveform generator is utilized to provide the original signal for the LIA, and the measuring results are shown in Figure 12.

Figure 12a illustrates the 0.4000 Vpp measuring results, in which the x-axis is the time, y-axis is the digital output, and the red dash lines are the maximum and minimal values of the output, respectively. The oscillation in Figure 12a is 24, corresponding with 0.01 mV for the 10 Vpp system. Furthermore, to illustrate the linear voltage response of the proposed LIA platform, the input signal ranging from 0 to 0.5 V with 0.1 mV step is implemented, and the measuring results are shown in Figure 12b, in which the x-axis and y-axis are the input analog signal and LIA digital output, respectively.

### 3.3. Spectrum Response

**Figure 13:**
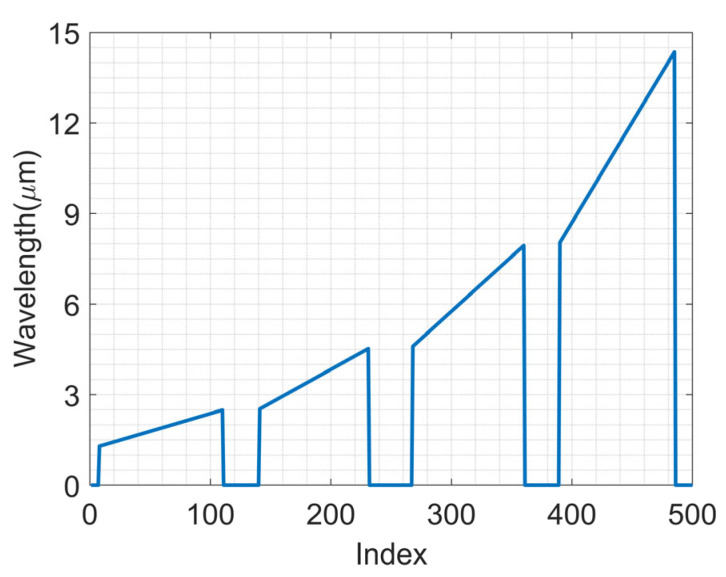
The wavelength-index of the CVF.

Since the LIA itself is not able to capture the optoelectrical detector spectrum response, the CVF is introduced. The wavelength–index relation of the CVF, which enables the optical wavelength ranging from 2.4 μm to 14 μm response, is drawn as follows. As shown in Figure 13, there are 500 points or index of the CVF, each of them corresponding with the given wavelength, then the link between LIA digital outputs and the spectrum wavelength.

**Figure 14:**
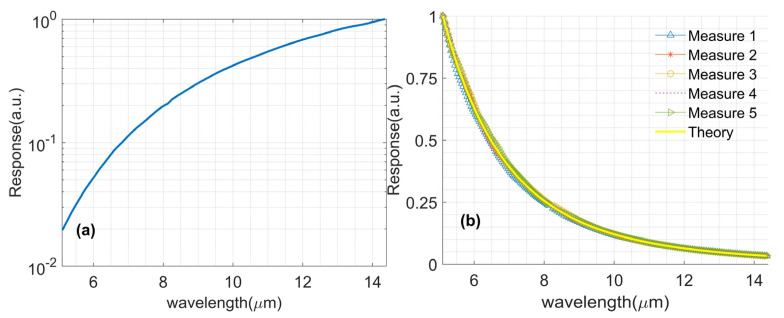
The results of the MCT experiments. (**a**) The spectrum response of MCT; (**b**) The blackbody spectrum measuring results.

With the setup shown in Figure 8, the temperature of the standard blackbody and the rate of the chopper are set as 50 °C and 800 Hz, respectively, and the results are exhibited in Figure 14. Figure 14a shows the MCT spectrum response at the given wavelengths, and Figure 14b shows the five measuring results with the NMSE are 0.9%, 0.46%, 0.59%, 0.84%, and 0.61%, respectively, which express the robust performance of the proposed LIA system.

**Figure 15:**
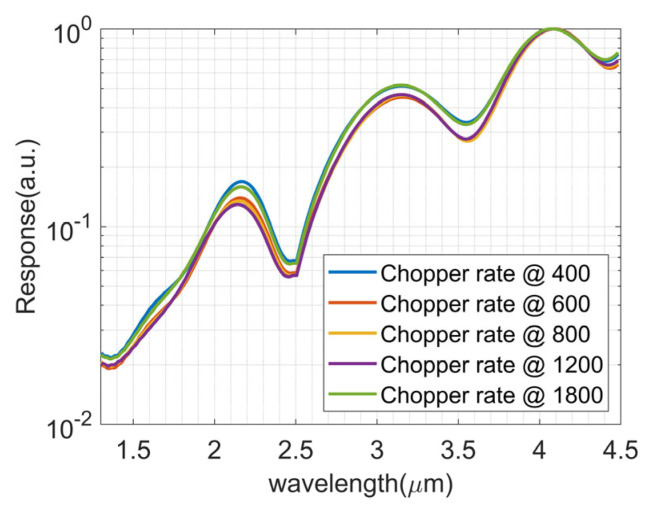
The responses of InSb at various chopper rates.

To analyze the influence of the chopper rate of the LIA, experiments at various chopper rates of 400 Hz, 600 Hz, 800 Hz, 1200 Hz, and 1800 Hz were conducted with the InSb detector. Since the InSb is sensitive at the spectrum for less than 6 μm, a blackboy at 1000 °C was implemented to complete the experiments, and the three measuring results with the NMSE are 0.602%, 0.722%, 0.629%, 0.815%, and 0.664%, as depicted in Figure 15, respectively.

## 4. Discussion

From the above results, even the chopper rate would affect the measuring results, especially in the relatively long wavelength part where the detector spectrum responses are similar. It is clear that the LIA proposed in this manuscript are sufficient to capture the spectrum response with choppers with various rates. Furthermore, since the results from lower chopper rates are much more stable, a low chopper rate should be set to capture the calibrating spectrum response.

## 5. Conclusions

In this paper, a digital implementation of the LIA is presented, which reduces the complexity and size of the LIA instrument, making it more practical for various applications. The digital LIA is designed and simulated using MATLAB, and experimental results demonstrate its effectiveness in accurately measuring the amplitude of the signals.

Furthermore, a setup is proposed for accurately capturing the optical spectrum from the black bodies, which utilizes a chopper, circular variable filter, and MCT/InSb detectors. The accuracy of the setup is demonstrated through experimental results.

Overall, this paper presents a comprehensive design and implementation of a digital LIA, as well as a practical setup for accurately measuring optical spectra. The proposed algorithm and design have potential applications in various fields, including optics, electronics, and signal processing.

## Figures and Tables

**Table 1:** Parameters for simulation.

Parameters (Unit)	Values
Magnitude (a. u.), A	7
Initial phase (rad), $\varphi_{s}$	π/3
Sampling frequency (kHz), $f_{clk}$	625
Angular speeds of original and reference signals (rad/s), $\omega_{s}$, $\omega_{r}$	2π × 1000
IIR Cutting-off frequency, $f_{c}$	100
Order of the IIR	4
SNR (dB)	−10

**Table 2:** The NMSE results of Phase error and amplitude captured in simulations.

NMSE of Phase	NMSE of Amplitude
0.18%	0.826%

**Table 3:** The parameters in experiments.

Parameters (Unit) or Resources	Values
Analog Voltage (Vpp), A	0.4000
SNR	−10
$\mathrm{Angular}\;\mathrm{speeds}\;\mathrm{of}\;\mathrm{original}\;\mathrm{and}\;\mathrm{reference}\;\mathrm{signals}\;(\mathrm{rad}/s),\;\omega_{s}$	2π×800
$\mathrm{Sampling}\;\mathrm{frequency}\;(\mathrm{kHz}),\;f_{clk}$	625
$\mathrm{IIR}\;\mathrm{Cutting}-\mathrm{off}\;\mathrm{frequency}\;(\mathrm{Hz}),\;f_{c}$	20
Order of the IIR	4
Lookup table	13,107
Embedded RAM	915
Multiplier DSP	20

## Data Availability

The data are available for reasonable request.
